# Intra- and Inter-Observer Reliability of ChatGPT-4o in Thyroid Nodule Ultrasound Feature Analysis Based on ACR TI-RADS: An Image-Based Study

**DOI:** 10.3390/diagnostics15202617

**Published:** 2025-10-17

**Authors:** Ziman Chen, Nonhlanhla Chambara, Shirley Yuk Wah Liu, Tom Chi Man Chow, Carol Man Sze Lai, Michael Tin Cheung Ying

**Affiliations:** 1 Department of Health Technology and Informatics, The Hong Kong Polytechnic University, Kowloon 999077, Hong Kong; 2 School of Healthcare Sciences, Cardiff University, Cardiff CF14 4XN, UK; 3 Department of Surgery, The Chinese University of Hong Kong, Prince of Wales Hospital, Shatin, New Territories 999077, Hong Kong

**Keywords:** large language model, ChatGPT, thyroid nodule, ultrasound features, observer agreement

## Abstract

**Background/Objectives:** Advances in large language models like ChatGPT-4o have extended their use to medical image analysis. Accurate assessment of thyroid nodule ultrasound features using ACR TI-RADS is crucial for clinical practice. This study aims to evaluate ChatGPT-4o’s intra-observer consistency and its agreement with an expert in analyzing these features from ultrasound image assessments based on ACR TI-RADS. **Methods:** This cross-sectional study used ultrasound images from 100 thyroid nodules collected prospectively between May 2019 and August 2021. Ultrasound images were analyzed by ChatGPT-4o, following ACR TI-RADS guidelines, to assess features of thyroid nodule including composition, echogenicity, shape, margin, and echogenic foci. The analysis was repeated after one week to evaluate intra-observer reliability. The ultrasound images were also analyzed by another ultrasound expert for the evaluation of inter-observer reliability. Agreement was measured using Cohen’s *Kappa* coefficient, and concordance rates were calculated based on alignment with the expert’s reference classifications. **Results:** Intra-observer agreement for ChatGPT-4o was moderate for composition (*Kappa* = 0.449) and echogenic foci (*Kappa* = 0.404), with substantial agreement for echogenicity (*Kappa* = 0.795). Agreement was notably low for shape (*Kappa* = −0.051) and margin (*Kappa* = 0.154). Inter-observer agreement between ChatGPT-4o and the expert was generally low, with *Kappa* values ranging from −0.006 to 0.238, the highest being for echogenic foci. Overall concordance rates between ChatGPT-4o and expert evaluations ranged from 46.6% to 48.2%, with the highest for shape (65%) and the lowest for echogenicity (29%). **Conclusions:** ChatGPT-4o showed favorable consistency in assessing some thyroid nodule features in intra-observer analysis, but notable variability in others. Inter-observer comparisons with expert evaluations revealed generally low agreement across all features, despite acceptable concordance for certain imaging characteristics. While promising for specific ultrasound features, ChatGPT-4o’s consistency and accuracy still vary significantly compared to expert assessments.

## 1. Introduction

Recent advancements in large language models (LLMs), such as ChatGPT-4, have significantly expanded their applications from natural language processing to include computer vision and multimodal data analysis [1,2,3]. These models excel in processing and integrating multiscale and multisource data due to their sophisticated data processing capabilities and deep learning algorithms. This progress highlights their potential in the medical field, where LLMs are increasingly utilized to enhance diagnostic accuracy, operational efficiency, and the development of personalized treatment plans [4,5,6]. Their integration into healthcare settings has demonstrated substantial promise, improving the analysis of extensive medical data, identifying complex patterns, and supporting clinical decision-making.

Thyroid nodules are common in clinical practice, and their accurate assessment is essential for determining appropriate management strategies [7,8]. The American College of Radiology Thyroid Imaging Reporting and Data System (ACR TI-RADS) provides a standardized framework for evaluating the ultrasound features of thyroid nodules [9]. This system includes comprehensive lexicons and definitions for aspects such as composition, echogenicity, shape, margin, and echogenic foci. While ACR TI-RADS aims to enhance diagnostic consistency and facilitate risk stratification, its effectiveness depends heavily on clinician expertise and can be labor-intensive [10,11,12]. The rapid advancement of artificial intelligence (AI) has provided new solutions for the objective analysis of thyroid nodules, ranging from studies on computer-aided diagnosis tools to the more recent exploration of LLMs [13,14,15,16]. Although previous research has primarily focused on leveraging LLMs for generating standardized ultrasound reports and aiding thyroid nodule diagnosis [5,17,18], their direct application in ultrasound image analysis remains underexplored. Our previous study evaluated the feasibility of LLMs in classifying thyroid nodules as benign or malignant based on ultrasound images, demonstrating their potential in malignancy differentiation [19]. However, that study did not assess their ability to analyze specific ultrasound features according to TI-RADS criteria.

This study aims to evaluate the performance of ChatGPT-4o in analyzing thyroid nodule ultrasound images following the ACR TI-RADS guidelines. Specifically, the research investigates the intra-observer consistency of ChatGPT-4o through repeated analyses and measures inter-observer agreement by comparing the model’s assessments with those of an experienced expert, whose assessments were considered as a benchmark for reference. The analysis encompasses various ultrasound features, including composition, echogenicity, shape, margin, and echogenic foci. By exploring the practical utility of ChatGPT-4o in clinical settings, this study aims to assess its reliability in enhancing diagnostic accuracy and consistency in medical image analysis, potentially reducing observer variability and improving workflow efficiency in thyroid nodule evaluation.

## 2. Materials and Methods

### 2.1. Ethical Statement and Informed Consent

This cross-sectional clinical study was conducted following approval from our institution’s Institutional Review Board of The Hong Kong Polytechnic University (Protocol code HSEARS20190123004. Approval date: 30 January 2019) and adhered to the ethical principles outlined in the Declaration of Helsinki. Prior to participation, written informed consent was obtained from all patients involved in the study.

### 2.2. Image Dataset

The image dataset for this study consisted of thyroid nodule ultrasound images prospectively collected from patients who underwent ultrasound examinations and subsequent pathological diagnoses at our institution between May 2019 and August 2021. Inclusion criteria required nodules to have a maximum diameter greater than 1 cm and clear imaging quality, without measurement markers. A total of 100 thyroid nodules meeting these criteria were randomly selected. Ultrasound examinations were conducted using the Aixplorer Ultrasound Imaging System (SuperSonic Imagine, Aix-en-Provence, France) equipped with a linear transducer (SL15-4, 4–15 MHz). Both longitudinal and transverse views of each nodule were captured and preserved for subsequent analysis. All procedures were performed by a sonographer with at least three years of clinical experience.

**Figure 1:**
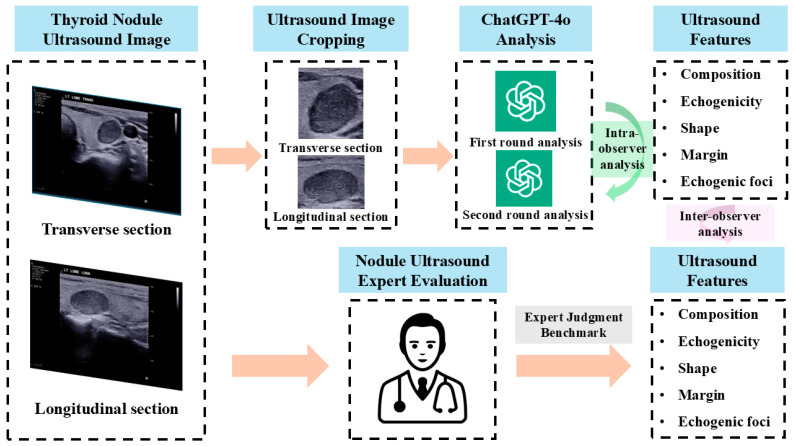
Workflow for Thyroid Nodule Ultrasound Feature Analysis Using ChatGPT-4o and Expert Evaluation. Transverse and longitudinal sections of thyroid nodule ultrasound images are cropped and analyzed by ChatGPT-4o. Intra-observer agreement is assessed between two rounds of ChatGPT-4o analyses. Inter-observer agreement is evaluated by comparing ChatGPT-4o’s assessments with expert evaluations, focusing on ultrasound features such as composition, echogenicity, shape, margin, and echogenic foci.

The study workflow is illustrated in Figure 1.

### 2.3. Large Language Model Analysis

ChatGPT-4o, an advanced LLM developed by OpenAI (San Francisco, CA, USA), was used for this research. As one of the latest iterations, ChatGPT-4o was trained on datasets updated through June 2024. To ensure analytical consistency, all operations were performed by the same, trained operator. All model parameters were maintained at their default settings provided by the OpenAI platform, with no modifications.

Ultrasound images of thyroid nodules, including transverse and longitudinal views, were uploaded to ChatGPT-4o. These images were not published online to prevent their use in pre-training the model. The images were pre-processed by cropping to include only the nodule and its surrounding thyroid tissue, eliminating extraneous information that could interfere with the model’s analysis. This step was essential for focusing the model’s attention on relevant features.

To ensure methodological consistency, a single standardized prompt with identical wording was applied in all evaluations without modification across sessions. Specifically, the following prompt was used in the analyses:

*“Please assume the role of an experienced ultrasound physician specializing in the diagnosis of thyroid nodules. I will present you with two ultrasound images of a thyroid nodule: the first image is a transverse view, and the second image is a longitudinal view. To ensure your focus is solely on analyzing the nodule’s characteristics, I have removed any non-essential information from the images that might interfere with your judgment, retaining only the nodule and its surrounding thyroid tissue. According to the ACR TI-RADS guidelines, please carefully evaluate and classify the ultrasound features of the nodule, considering the following aspects:*

1. *Composition: cystic or almost completely cystic, spongiform, mixed cystic and solid, solid or almost completely solid.*
2. *Echogenicity: anechoic, hyperechoic or isoechoic, hypoechoic, very hypoechoic.*
3. *Shape: taller-than-wide, wider-than-tall.*
4. *Margin: smooth, ill-defined, irregular or lobulated, extrathyroidal extension.*
5. *Echogenic foci: none, large comet-tail artifacts, macrocalcifications, peripheral or rim calcifications, punctate echogenic foci.”*

ChatGPT-4o automatically analyzed the uploaded ultrasound images of thyroid nodules, assessing features according to the ACR TI-RADS guidelines. The evaluation encompassed various aspects, including composition (cystic, almost completely cystic, spongiform, mixed cystic and solid, solid or almost completely solid), echogenicity (anechoic, hyperechoic or isoechoic, hypoechoic, very hypoechoic), shape (taller-than-wide, wider-than-tall), margin (smooth, ill-defined, irregular or lobulated, extrathyroidal extension), and echogenic foci (none, large comet-tail artifacts, macrocalcifications, peripheral or rim calcifications, punctate echogenic foci). To maintain the independence of each analysis session, a new chat interface was used for each image set.

The operator repeated the analyses one week later using the same methodology to assess the intra-observer agreement of ChatGPT-4o.

### 2.4. Benchmark Evaluation

An expert in thyroid ultrasound, who is a board-certified radiologist with more than twenty years of clinical experience, independently reviewed all ultrasound images. The expert analyzed the images and classified the ultrasound features of the nodules based on the ACR TI-RADS guidelines, providing a benchmark for comparison. The expert’s evaluations were used as the benchmark for assessing the performance of ChatGPT-4o.

### 2.5. Statistical Analysis

The analysis of data was carried out with SPSS 26.0 (SPSS Inc., Chicago, IL, USA) and R statistical software, version 4.2.0 (R Foundation for Statistical Computing, Vienna, Austria). The intra-observer agreement of ChatGPT-4o’s analyses and the inter-observer agreement between ChatGPT-4o and the expert’s evaluations were assessed using Cohen’s *Kappa* coefficient. The *Kappa* statistic interpretation ranges were as follows: <0 (none agreement), 0–0.2 (slight agreement), 0.2–0.4 (fair agreement), 0.4–0.6 (moderate agreement), 0.6–0.8 (substantial agreement), and 0.8–1.0 (almost perfect agreement). Additionally, the concordance rate was calculated as the percentage of ultrasound features classified by ChatGPT-4o that were consistent with the expert’s reference classifications.

## 3. Results

### 3.1. Baseline Characteristics of the Image Dataset

The image dataset included a total of 100 thyroid nodules, of which 70 were classified as benign and 30 as malignant. The mean nodule size was 2.57 ± 1.23 cm. These nodules were derived from 98 patients, with a mean age of 54.26 ± 12.19 years, comprising 19 males and 79 females. A detailed summary of the baseline characteristics is provided in Table 1.

### 3.2. Intra- and Inter-Observer Agreement in Ultrasound Feature Assessment

The intra-observer agreement of ChatGPT-4o varied across different ultrasound feature categories. For composition, moderate agreement was observed (*Kappa* = 0.449). Echogenicity showed substantial agreement (*Kappa* = 0.795), while shape demonstrated no agreement (*Kappa* = −0.051). Margins exhibited slight agreement (*Kappa* = 0.154), and echogenic foci showed moderate agreement (*Kappa* = 0.404).

Similarly, the inter-observer agreement between ChatGPT-4o and the ultrasound expert varied across feature categories in both rounds. Composition demonstrated slight agreement (*Kappa* = 0.092 in the first round and 0.075 in the second round). Echogenicity showed no agreement (*Kappa* = −0.006 in the first round and −0.001 in the second round). Shape showed slight agreement (*Kappa* = 0.026 in the first round and 0.082 in the second round). Margins also exhibited slight agreement (*Kappa* = 0.096 in the first round and 0.092 in the second round). Echogenic foci demonstrated slight to fair agreement (*Kappa* = 0.142 in the first round and 0.238 in the second round).

**Figure 2:**
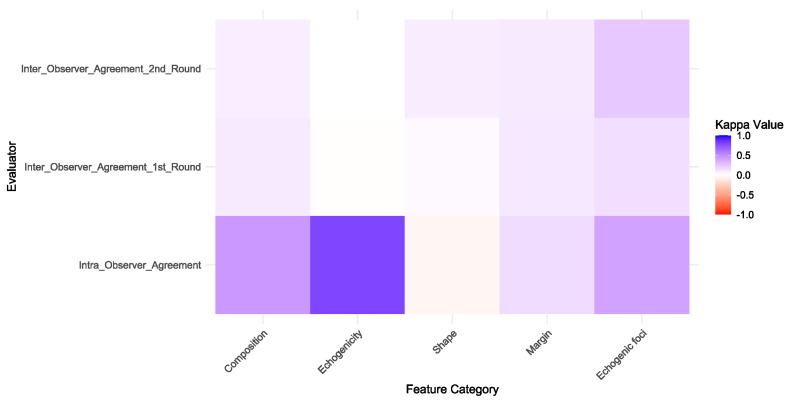
Heatmap of Kappa Values for Intra- and Inter-Observer Agreement in Ultrasound Feature Assessment. The heatmap illustrates the Kappa values for intra-observer and inter-observer agreement across different ultrasound feature categories. Kappa values are color-coded on a gradient from red (indicating very poor agreement) to blue (indicating excellent agreement), with a white transition in between. The gradient from red to white denotes poor agreement, whereas the transition from white to blue reflects improving agreement. Categories include Composition, Echogenicity, Shape, Margin, and Echogenic foci.

The details of the intra- and inter-observer agreement are summarized in Table 2 and illustrated in Figure 2.

### 3.3. Concordance Rates Between ChatGPT-4o and Expert Evaluations

**Figure 3:**
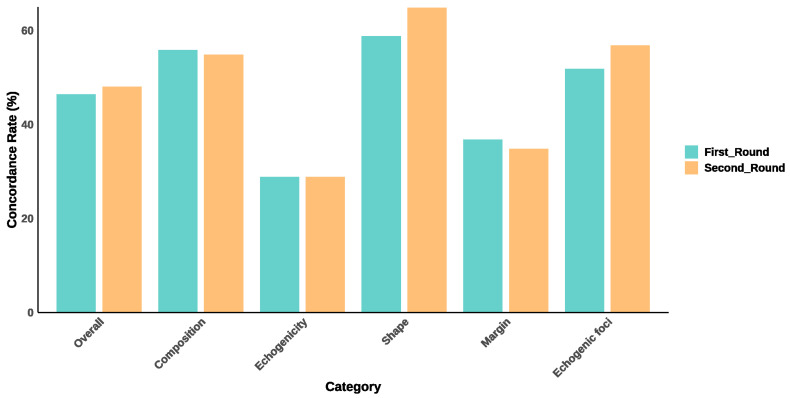
Concordance Rates between ChatGPT-4o and Expert Evaluations across Ultrasound Features. This bar chart illustrates the concordance rates between ChatGPT-4o and expert evaluations for various ultrasound features of thyroid nodules. The results are presented for two evaluation rounds.

The overall concordance rate was 46.6% in the first round and 48.2% in the second round, as shown in Table 3 and Figure 3. For specific feature categories, composition exhibited moderate concordance, with rates of 56.0% in the first round and 55.0% in the second round. Echogenicity showed the lowest concordance, remaining at 29.0% in both rounds. Shape demonstrated the highest concordance among all categories, with rates of 59.0% in the first round and 65.0% in the second round. Margin had relatively low concordance rates, with 37.0% in the first round and 35.0% in the second round. Lastly, echogenic foci showed fair concordance, with rates of 52.0% in the first round and 57.0% in the second round.

## 4. Discussion

The present study provides a comprehensive evaluation of the intra- and inter-observer consistency of ChatGPT-4o in analyzing thyroid nodule ultrasound features from image-based assessments according to ACR TI-RADS. Our findings indicate that ChatGPT-4o demonstrates moderate to substantial intra-observer agreement for features such as echogenicity and composition, reflecting reliable consistency in repeated image analyses. However, certain features like shape and margin exhibited considerably lower intra-observer consistency, indicating variability in the model’s performance. Furthermore, inter-observer agreement between ChatGPT-4o and the expert was generally low, with slight agreement across most categories, though concordance rates were acceptable for some feature categories.

Several studies have investigated the consistency of LLMs, such as ChatGPT, in medical applications. However, most of these studies were focused on text-based analysis rather than direct image interpretation. Jiang et al. assessed ChatGPT-4’s ability to classify thyroid nodules based on the ACR TI-RADS using structured ultrasound report data [20]. Their findings showed moderate intra-observer agreement, with an intraclass correlation coefficient of 0.732, indicating that LLMs can reliably classify thyroid nodules using standardized medical reports. In contrast, our study observed varying levels of intra-observer agreement across different ultrasound features. For instance, composition demonstrated moderate agreement (*Kappa* = 0.449), while echogenicity showed substantial agreement (*Kappa* = 0.795). However, no agreement was observed for shape (*Kappa* = −0.051), and margins had only slight agreement (*Kappa* = 0.154). The discrepancy between our findings and those of Jiang et al. likely arises from the different scopes of each study. While Jiang et al. focused exclusively on TI-RADS classifications, our study conducted a more granular analysis of specific ultrasound features, revealing areas where LLM performance may require further refinement. Additionally, Jiang et al. evaluated ChatGPT’s capacity to interpret structured data, whereas our study tested its ability to directly analyze ultrasound images. This distinction highlights the current limitations of LLMs, such as ChatGPT, in image interpretation, underscoring the need for further advancements in this area, which is also consistent with findings by Brin et al. [21] and Reith TP et al. [22], who observed significant variation in ChatGPT-4’s performance across different imaging modalities and noted its current unreliability for standalone clinical use in radiology. This inconsistency was particularly evident in the assessment of shape and margin features, which showed notably lower reproducibility compared with other ultrasound characteristics. For shape assessment, we speculate that this may result from the model’s inability to perform geometric measurements and its limited spatial perception. Determining whether a nodule is taller-than-wide requires comparing vertical and horizontal dimensions, which is challenging without true geometric measurement capability and sufficient spatial perception [23]. For the margin feature, accurate delineation of the interface between the nodule and surrounding thyroid tissue is essential. However, current LLM-based vision models are not specifically designed for medical imaging tasks and may favor global texture or semantic cues over fine boundary delineation, making them prone to errors when margins are blurred or irregular [24].

Our study also revealed considerable variability in inter-observer agreement between ChatGPT-4o and an ultrasound expert across various ultrasound features. For example, composition showed slight agreement between the two observers (*Kappa* = 0.092 in the first round and 0.075 in the second), and no agreement was found for echogenicity (*Kappa* = −0.006 in the first round and −0.001 in the second). Shape exhibited slight agreement (*Kappa* = 0.026 in the first round and 0.082 in the second), and margins similarly displayed slight agreement (*Kappa* = 0.096 in the first round and 0.092 in the second). These results suggest that LLMs like ChatGPT still face significant challenges in consistently interpreting more nuanced ultrasound features when compared to expert evaluations. The lack of agreement in categories such as echogenicity and margins likely reflect the complexity of these features, which often require subtle clinical judgment that current LLMs may not yet fully replicate. Similar findings were reported by Sievert et al., who examined ChatGPT’s performance in risk stratifying thyroid nodules based on text-based ultrasound reports using ACR TI-RADS [25]. Their study found a low overall agreement of 42% between ChatGPT and human evaluators. In contrast, our study observed slightly higher concordance rates between ChatGPT-4o and expert evaluations, with overall agreement rates of 46.6% in the first round and 48.2% in the second. The discrepancies in inter-observer agreement found in both our study and Sievert et al.’s work emphasize the need for further optimization of LLMs for clinical use. While moderate concordance rates were observed for certain ultrasound features, such as shape and composition, the overall lack of reliability in interpreting features like echogenicity and margins indicates that LLMs are not yet ready for clinical decision-making based on image interpretation.

When assessing inter-observer agreement between ChatGPT-4o and the expert, ChatGPT-4o demonstrated moderate to high concordance rates across most ultrasound feature categories (35.0–65.0%). However, the observed low or even negligible *Kappa* values (0.026–0.238) indicate poor consistency, which seems counterintuitive at first glance. This discrepancy arises from fundamental differences in how concordance rate and *Kappa* statistics assess agreement [26,27]. While concordance rate simply quantifies the proportion of matching classifications without adjusting for chance agreement, *Kappa* accounts for expected agreement due to randomness, making it a more rigorous metric for true consistency. In present study, one major explanation for this phenomenon lies in the imbalance of feature classifications, which distorts the expected agreement calculation [28]. For example, in ChatGPT-4o’s analysis, 85–86% of nodules were classified as “solid or almost completely solid” in composition, and 97–98% as “hypoechoic” in echogenicity. Similarly, in expert evaluations, 83% of nodules were categorized as “wider-than-tall” in shape. Given the predominance of these categories, a high observed concordance can occur even under random conditions, artificially inflating the expected agreement and thereby diminishing the *Kappa* value. Since the majority of nodules belong to a single category, the expected agreement by chance is already high, leading to a disproportionately low *Kappa* value despite a moderate concordance rate. This finding underscores an important limitation: while concordance rate provides a straightforward measure of agreement, it may overestimate model reliability when class distributions are highly skewed. In contrast, *Kappa* offers a more stringent evaluation of consistency, which is essential for assessing the reproducibility of AI-driven diagnostic tools in clinical applications.

At present, LLM-based systems may be more appropriately positioned as supportive tools for education, training, or reducing observer variability rather than for direct clinical decision-making. In clinical practice, diagnostic tools are required to demonstrate high levels of reliability and safety prior to implementation. Misclassification of key nodule features could lead to inappropriate TI-RADS categorization, thereby influencing decisions regarding fine-needle aspiration or follow-up. Although ChatGPT-4o achieved moderate reproducibility for certain ultrasound features, the overall low inter-observer agreement with expert assessments indicates that the model is not yet suitable for independent clinical application. Clinical deployment will necessitate performance thresholds comparable to those of experienced radiologists, supported by large-scale validation, to ensure patient safety and diagnostic reliability.

Our study has limitations that warrant consideration. First, ChatGPT-4o is primarily designed for text-based tasks, and its ability to directly interpret images, particularly complex ultrasound features, is limited. Future research should emphasize the innovation and adaptation of LLMs specifically for medical image analysis to enhance their effectiveness in this domain. Second, the ultrasound images analyzed were derived from a controlled dataset, which may not fully capture the diversity of images encountered in clinical practice, potentially limiting the generalizability of our results. Third, although all images were acquired prospectively using standardized scanning protocols by experienced sonographers, variability in image quality may still have influenced the results. Factors such as contrast, spatial resolution, artifacts, and subtle differences in acquisition techniques can affect the visibility and interpretation of key ultrasound features. This limitation is particularly important for AI-driven image analysis, as models may be more sensitive to such variability than human observers. Future studies should therefore incorporate systematic assessments of these image quality factors to better understand their impact on model performance. Finally, although a single standardized prompt with identical wording was applied in all evaluations to minimize variability, different prompt phrasings could potentially influence model performance, and this remains an important aspect for future investigation.

## 5. Conclusions

While ChatGPT-4o demonstrates moderate to substantial intra-observer reproducibility in analyzing thyroid nodule ultrasound features from medical image assessments according to ACR TI-RADS, significant variability remains, particularly in features such as shape and margin. This inconsistency is observed not only in intra-observer analyses but also in inter-observer comparisons with expert assessments, where agreement was generally low. Despite its potential as a supportive tool for medical image analysis in clinical settings, ChatGPT-4o requires further refinement to improve its reliability across all ultrasound features. Enhancing the model’s performance through additional validation and optimization of its medical image interpretation capabilities is essential for its successful and consistent integration into clinical practice.

## Figures and Tables

**Table 1:** Baseline characteristics of image dataset.

Characteristic	Statistics
Patients	98
Sex (Male/Female)	19/79
Age (years)	54.26 ± 12.19
Nodules (Benign/Malignant)	100 (70/30)
Nodule size (cm)	2.57 ± 1.23

Notes: Categorical variables are presented as numbers, and continuous variables as mean ± standard deviation.

**Table 2:** Intra- and inter-observer agreement in ultrasound feature assessment of thyroid nodules by ChatGPT-4o and ultrasound expert.

Category	ChatGPT-4o		*Kappa*	Ultrasound Expert	*Kappa* ^#^	*Kappa* *
	1st Round	2nd Round				
**Composition**			0.449		0.092	0.075
cystic or almost completely cystic	2	3		0		
spongiform	0	0		8		
mixed cystic and solid	13	11		37		
solid or almost completely solid	85	86		55		
**Echogenicity**			0.795		−0.006	−0.001
anechoic	2	3		0		
hyperechoic or isoechoic	0	0		69		
hypoechoic	98	97		30		
very hypoechoic	0	0		1		
**Shape**			−0.051		0.026	0.082
wider-than-tall	62	68		83		
taller-than-wide	38	32		17		
**Margin**			0.154		0.096	0.092
smooth	52	43		26		
ill-defined	23	22		61		
lobulated or irregular	25	35		11		
extra-thyroidal extension	0	0		2		
**Echogenic foci**			0.404		0.142	0.238
none	54	52		58		
large comet-tail artifacts	0	0		0		
macrocalcifications	1	0		13		
peripheral (rim) calcifications	0	0		1		
punctate echogenic foci	45	48		28		

Notes: *Kappa* denotes intra-observer agreement for ChatGPT-4o. *Kappa* ^#^ and *Kappa* * indicate inter-observer agreements between the first and second rounds of ChatGPT-4o, respectively, and the ultrasound expert. Bolded terms (Composition, Echogenicity, Shape, Margin, and Echogenic foci) represent the five major ultrasound feature categories according to the ACR TI-RADS classification.

**Table 3:** Concordance rates between ChatGPT-4o and expert evaluations of thyroid nodule ultrasound features.

Category	1st Round Concordance Rate	2nd Round Concordance Rate
Overall	46.6%	48.2%
Composition	56.0%	55.0%
Echogenicity	29.0%	29.0%
Shape	59.0%	65.0%
Margin	37.0%	35.0%
Echogenic foci	52.0%	57.0%

## Data Availability

The data presented in this study are available from the corresponding author upon reasonable request. Data is not publicly available due to privacy or ethical concerns.
